# English proficiency as a performance of digital social capital: understanding how Chinese study abroad students use WeChat for the symbolic purpose of English language learning

**DOI:** 10.1186/s40711-022-00177-y

**Published:** 2022-11-25

**Authors:** Jordan Carolan

**Affiliations:** 1 University College Cork, College Road, Cork, Ireland; 2 Navan, Ireland

**Keywords:** Digital social capital, Symbolic interactionism, WeChat, Chinese EFL learners

## Abstract

This study investigates how Chinese study abroad students utilize WeChat for the symbolic purpose of English language learning while exploring what particular features of WeChat are beneficial to one’s English learning. It also explores how English proficiency acts as a form of digital social capital in China, with a particular focus on how WeChat acts as a stage from which users can perform their perceived higher-social class. By deploying a symbolic interactionist approach and conceptualizing an appropriate theoretical framework, this study aims to determine whether students fully engage with WeChat’s symbolic meaning as an English learning tool. Qualitative methods of research consisting of semi-structured interviews and a walkthrough of WeChat are carried out which investigates how English learning features are accessed on WeChat and how they ultimately shape learners’ symbolic meanings of WeChat. It is found that performing high English proficiency on WeChat is associated with negative connotations (bragging) due to links between English level and class background. Moreover, factors such as filial piety prevented users from performing their English proficiency and fully engaging with WeChat as a learning tool also.

## Introduction

This study engages the topic of English proficiency as a performance of digital social capital and aims to understand how the Chinese social media app WeChat impacts English language learning among Chinese study abroad (SA) students in Ireland. Moreover, it investigates whether Chinese SA students attach symbolic meaning to WeChat as a potential English as a foreign language (EFL) learning tool.

WeChat is China’s largest social media app, with an estimated one billion monthly users. Furthermore, regarding access to technology, in 2018, around 50 percent of the Chinese population used a smartphone, and by the end of 2020, about 989 million people had access to the Internet within China (Thomala 2021). Meanwhile, there are an estimated 400 million Chinese EFL learners, with College English (CE) being compulsory until the second year of university and English representing a compulsory element of the university entrance exam (Bolton and Graddol 2012; Cheng and Dong 2017). These figures demonstrate the vast scale of WeChat users, EFL learners, and Internet connectivity within China and, therefore, indicate WeChat’s potential to aid EFL learners if its functions are used accordingly.

WeChat was selected for this particular study because it is the largest social media app in China. Moreover, while other Chinese social media apps such as QQ have been analyzed in relation to assisting EFL learning (Lei et al. 2018; Li and Zhou 2021), 77% of Internet users used WeChat in the third quarter of 2021, compared with 61.5% who had used QQ (Statista 2022). Furthermore, a greater volume of recent research concerning EFL learning has been conducted on WeChat compared with QQ or any other social media app in China (Wang and Crosthwaite 2021; Namaziandost et al. 2021; Zhang et al. 2021).

However, despite the fact that more recent research has been conducted on WeChat, Hou et al. (2020: 1814) state there are currently “few studies on the relationship between WeChat and learning from university students’ perspective,” and even fewer studies that focus solely on EFL learning on WeChat in relation to SA students in particular. Therefore, the results of this study may assist in furthering the understanding of how WeChat can be used to aid Chinese EFL students more effectively in the future.

Moreover, this study focuses on online sociality, analyzing user agency and applying it to EFL learning. van Dijck (2013) states that explicit users refer to real or actual users that interact with social media, and these users can be within a particular demographic. Explicit users may also be used as ethnographic subjects and thus be interviewed about their experiences. Therefore, van Dijck’s (2013) notion of explicit users is highly influential in regard to choosing participants for this particular study. Furthermore, as this study employs a symbolic interactionist approach, participant findings will be analyzed in conjunction with Light, Burgess, and Duguay’s (2018) app walkthrough method in order to highlight how the objective meaning of WeChat as a social media app can shift to the symbolic meaning of WeChat as a potential EFL learning tool. In regard to analyzing the findings, there will be a particular focus on Goffman’s presentation of self, as well as Bourdieu’s premise of social capital, which has been reconceptualized in the form of Julien’s (2015) concept of digital social capital in order to be applicable to WeChat and modern-day technology.

This study has practical implications in determining whether WeChat has beneficial features in regard to EFL learning. Furthermore, this article will highlight why EFL learners may not engage in WeChat’s symbolic meaning as an EFL learning tool and subsequently provide suggestions on how to potentially improve learners’ engagement.

## Literature review

### App walkthrough: introducing the WeChat app

While discussing WeChat’s design and its potential as a language learning application, Lei and Liu (2020: 2) state that WeChat has 18 functions in total and indicate that “WeChat is a lightweight application with great convenience, good experience, rich functions and simple development, which has its unique advantages for mobile learning platform development.” While Lei and Liu (2020) are some of the few researchers to briefly list the functions that can potentially assist with language learning, they do not specify how exactly WeChat can assist learners or how effective WeChat’s respective functions are. Furthermore, as will be mentioned in more detail in the proceeding section, previous research (Cheng and Dong 2017; Shi et al. 2017; Wang and Crosthwaite 2021) only focused on the instant messaging aspect of WeChat. Moreover, Zhang et al. (2021) explored the effectiveness of the WeChat official account’s tweet-based writing. However, while Chen and Wang (2022) conducted a small-scale app walkthrough of a singular subscription account on WeChat, no thorough app walkthrough of the effectiveness of WeChat’s functions for EFL learning has yet been undertaken.

As the functions of WeChat which may assist with English learning have not yet been thoroughly explored, the app walkthrough method devised by Light, Burgess, and Duguay (2018: 882) will be undertaken. It involves “engaging directly with an app’s interface to examine its technological mechanisms and embedded cultural references to understand how it guides users and shapes their experiences.” While this study will pioneer the use of an app walkthrough regarding comprehensively detailing how WeChat may assist with EFL learning, van Dijck’s (2013) theory of sociality and explicit users means that conducting qualitative interviews with Chinese SA students as ethnographic subjects will subsequently complement and verify the findings of the walkthrough method in order to answer the research questions more comprehensively.

### Chinese students’ attitudes toward WeChat in facilitating English learning

An increasing amount of research explores how social media applications can assist with EFL learning. Maulina et al. (2022) state that social media apps have advantages regarding ease of use, fast connectivity, and low costs. However, as mentioned previously, few studies have systematically explored WeChat’s effectiveness in facilitating English learning in particular. Therefore, it is essential to determine whether users are open to engaging with WeChat as an EFL tool, considering that WeChat is objectively a social media application. Moreover, little research has yet been conducted on Chinese SA students’ use of WeChat as a learning tool, as the previous literature has so far only focused on foreign and mixed second-language learners of the Chinese language (Jin 2018; Chen and Zhan 2020).

While research on Chinese SA students is limited, the study undertaken by Hou et al. (2020), while not focusing specifically on EFL, investigated how university students perceive the role of WeChat in facilitating learning. They found that although WeChat presents many learning benefits, self-control is an essential factor regarding WeChat being used successfully in the learning context.

Moreover, regarding the previous literature that touched upon WeChat’s potential for English learning, Shi et al. (2017) found that students’ English proficiency and academic performance improved after using WeChat’s instant messaging feature as an EFL learning tool. Furthermore, unlike the previous studies mentioned which did not focus solely on WeChat’s effect on EFL learning, Cheng and Dong (2017) found that 95% of their interviewees perceived a class WeChat group chat as useful for English learning. Within the aforementioned group chat feature, teachers could share English articles, and students were allowed to have an open dialog and pose questions to their teachers. However, despite WeChat’s potential benefits to learning, a large body of research portrays the everyday use of WeChat as a learning distraction (Chen 2018; Huang 2019; Lei and Cai 2020). Therefore, under symbolic interactionism, determining whether students are willing to “participate in the representation process” of WeChat as a learning app remains to be seen (Segre 2019: 380).

From a symbolic interactionist perspective, an emphasis is placed on an agent’s interpretation of symbolic meanings and the self, which are “formed, sustained, weakened and transformed in their interaction” (Blumer 1969: 21; Lee 2014). The meaning that users attach to WeChat is influenced by outside and societal factors that subsequently shape users’ attitudes and how they perceive and use the app. Furthermore, this also influences how effective an app is in achieving its respective outcomes (Chen et al. 2020). According to Chen et al. (2020), technology represents both objective and symbolic meaning; with objective meaning relating to the inherent features of the technology and whether they are used to produce an effective outcome, while symbolic meaning relates to the subjective and social interpretations of a particular technology (Markus and Silver 2008; Jung and Lyytinen 2014; Cecez-Kecmanovic et al. 2014). Therefore, this study will address a gap in research by exploring the symbolic meanings that Chinese SA students attach to the objective functions of WeChat concerning EFL learning.

### The intersection of English learning and social capital in China

Liu and Chiang (2019) found that language subjects such as English are more associated with a higher-class background than other subjects within China. Moreover, Yang (2007: 12) argued that the use of the English language in an online context is portrayed as “showing off” within China due to the links between English proficiency and wealth; however, Yang’s (2007) study lacked theoretical underpinnings and focused only on the use of English on electronic bulletin boards. Therefore, to systematically analyze the links between social class and English proficiency, Gao (2014) incorporated Bourdieu’s theory of social capital and suggested that English education is a site for social class differences. Students can improve their English proficiency to gain a competitive advantage by attending higher-quality schools or private English classes with higher expenses, which consequently excludes individuals with lower social and economic capital.

Therefore, inspired by Gao’s (2014) study, this article also uses a variation of Bourdieu’s theory of social capital. In order to understand what social capital is, Bourdieu and Richardson (1986: 248) state that social capital is the “aggregate of the actual or potential resources” which are linked to “membership in a group.” In this article, the group being discussed is Chinese EFL learners. As the English language represents a compulsory element of education and is a “credential” deemed as “necessary” by Chinese employers (Kubota 2015: 3), this group has institutional support that reproduces lasting relationships and symbolic benefits (Bourdieu and Richardson 1986: 249).

This analysis of the links between English proficiency and social capital is relevant to studying WeChat as an EFL learning tool. As a social media application, any actions or performances of the self on the app will be seen by observers and will result in social connotations for the users, which may affect their perceived symbolic meaning and subsequent usage of WeChat (Jones and Volpe 2011).

However, while social capital is a classical sociological theory that arose before the digital age of social media, it is, therefore, necessary to integrate an updated interpretation of social capital that can adequately explain the prestige associated with performing one’s English proficiency on WeChat. Julien (2015: 365), therefore, conceptualizes the notion of “digital social capital,” which refers to the expressions of social capital that arise in online contexts which affect and extend one’s social relationships. Julien (2015) states that digital social capital deviates from Bourdieu’s original interpretation; however, it is possible to convert digital social capital to social capital in the physical world if the entities which the agent knows in the physical world are also familiar with online culture and perceive digital social capital accumulation positively. Furthermore, a social hierarchy exists in the online world, and online posts are assigned digital social capital, resulting in recognition and distinction not only among disparate social groups but also between agents within the same social group (Julien 2015). However, while Julien’s (2015) study analyzed Internet memes only, this study will pioneer the exploration of English proficiency (in the form of text, pictures, and status updates) as a form of digital social capital and how this affects users’ engagement with WeChat in regard to English learning.

### English education as a dramatization of oneself

Due to the unique context of EFL learning via a social media application, Chinese SA students who use WeChat can easily project an image of themselves online as a performance of their true selves. This study will use Goffman’s (1959) theory of presentation of self, and despite representing a classical theory, Hogan (2010: 377) states that the theory is “becoming increasingly popular as a means for explaining differences in meaning and activity of online participation.” Case in point, Goffman’s (1959) presentation of self has been applied to studies on the self for various social media apps in the modern day (Zhang 2022; Schibblock et al. 2022).

Goffman’s (1959: 22) theory places pertinence on “performances,” which refers to “an activity of an individual which occurs during a period marked by his continuous presence before a particular set of observers and which has some influence on the observers.” Moreover, regarding social media, Hogan (2010: 378) states that agents will predominantly attempt to perform an “idealized” and positive version of themselves while in view of observers. Furthermore, Huang et al. (2020) have used the theory of presentation of self in conjunction with Chinese college students and WeChat in particular, and it was found that students both hid and altered certain WeChat moments as they feared potential negative comments from family members. On the other hand, moments that demonstrated academic/professional progress were purposely shown to employers to make the user appear more impressive.

Due to the Confucian background of the interviewees in this study, maintaining strong family connections and obeying one’s elders is a pertinent feature of Confucian cultures, which may affect how the participants perform their identities on WeChat (Wong 2014; Wang 2021). As the findings of Huang et al. (2020) have shown, one’s relatives affect the user’s symbolic meaning of WeChat and influence how one presents the self. However, considering that the participants are Chinese SA students who are often portrayed as “neoliberal subjects” (Xu 2022: 153), it is unclear what effect traditional Confucian values will have on this group, considering that those with neoliberal characteristics are individualistic and exhibit generational tension (Kubota 2011; McGuigan 2014).

The aims of this research are underpinned by the literature on symbolic interactionism as Jones and Volpe (2011: 426) stated that one’s social networks have a direct influence on how one performs the self and that one’s social networks “generate meaning” for the user. Moreover, Chen et al. (2020: 4) found that symbolic meaning is equally as “important as the innate technical features and capabilities of a particular app” and that other user’s accounts and presentations of self can affect a user’s interpretation of the symbolic meaning and use of technology.

In Goffman’s (1959: 55) theory of presentation of self, the comparison of a nurse working in a hospital was used to “dramatize” one’s work, with a nurse only appearing “impressive” if they are physically performing their role for others to see. This same principle also applies to English learning: whether Chinese SA students perform their digital social capital online in the form of publicly sharing posts, materials, and photos related to EFL learning on WeChat in order to be regarded as impressive in the eyes of others. On the other hand, Chinese SA students may also avoid publicly performing their digital social capital in order to prevent negative connotations of bragging and links with wealth (Yang 2007).

In regard to why the presentation of self is relevant to a study exploring the symbolic effectiveness of WeChat as an English learning tool, it is pertinent to take into account that WeChat’s objective symbolism is as a social media app. Therefore, users will inevitably perform the self in front of others in some capacity if they use WeChat for the symbolic purpose of English learning. This article aims to determine whether the social connotations of WeChat as a social media app subsequently affect users’ engagement with WeChat for the symbolic purpose of learning English.

**Fig. 1:**
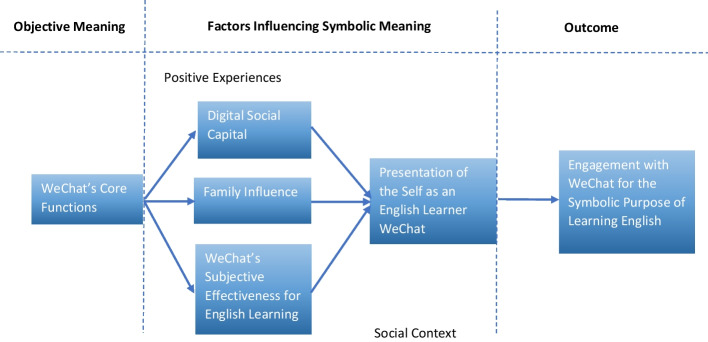
Theoretical framework for examining WeChat as an EFL learning tool

According to McCay-Peet and Quan-Haase (2016) on symbolic interactionism, the presentation of self directly affects one’s engagement with an app for a particular purpose. This is pertinent, as whether the participants’ usage of WeChat for the symbolic purpose of EFL learning is perceived positively will determine how likely they are to perform themselves and ultimately engage with WeChat fully for EFL learning. Engagement entails the “quality of user experience with technology” (McCay-Peet and Quan-Haase 2016: 200). Therefore, in order to determine whether the participants fully engage with WeChat’s symbolic meaning as an English learning tool, the theoretical framework of this study was conceptualized and is shown in Fig. 1.

1. *WeChat’s Core Functions* The objective functions of a freshly downloaded version of WeChat
2. *Digital Social Capital* Whether users associate WeChat’s symbolic meaning as an English learning tool with digital social capital
3. *Family Influence* Whether users’ family influence their usage of WeChat for the symbolic purpose of English learning
4. *WeChat’s Subjective Effectiveness for English Learning* Whether users report that WeChat’s objective functions are useful for the symbolic purpose of English learning. Considering that WeChat’s objective meaning is as a social media app by default, any user who uses WeChat or one of its functions for English language learning is attaching a symbolic meaning to the application/particular function
5. *Positive Experiences* Whether the users’ perceptions of WeChat’s features, digital social capital, family influence, and performance of the self are positive
6. *Social Context* The size and nature of the social networks that users engage with (McCay-Peet and Quan-Haase 2016)
7. *Presentation of the Self* Influenced by the previous factors, whether users can freely perform their identity as an English language learner on WeChat in order to fully engage with WeChat for English learning
8. *Engagement* The quality of users’ experiences and level of interaction with WeChat for the symbolic purpose of learning English

## Research methods

### Research questions

This research was guided by a number of research questions/sub-questions:

1. What features of WeChat may assist Chinese study abroad students in regard to English learning?
  1a. Do Chinese study abroad students engage with WeChat’s symbolic meaning as an English learning tool?
2. Is English proficiency portrayed as a form of digital social capital on WeChat?

### Qualitative approach

By implementing a qualitative approach, this study provides an in-depth and interpreted understanding of the interviewees by learning about their social and material circumstances, experiences, perspectives, and histories (Salmons 2016). Within this approach, an individual’s words and behaviors are analyzed, and researchers must set aside their own biases and accurately represent the interviewee within their research (Taylor et al. 2015).

This study combines both inductive and deductive elements. Firstly, as Taylor et al. (2015) note, qualitative data are inherently inductive because understandings, concepts, and insights are collected from empirical data. Secondly, the study contains deductive reasoning, as during the literature review stage, previous research was analyzed, which led to the formation of the research questions.

In order to answer the respective research questions, semi-structured interviews were conducted with Chinese SA students in the 20–23 age range within an Irish university. Links were subsequently drawn, and research was conducted in this area that previous studies have not yet addressed, such as the presentation of oneself and students’ perceptions and attitudes toward posting one’s English language learning experiences on WeChat. As a deeper understanding of students’ experiences and attitudes is required, qualitative data in the form of semi-structured interviews were necessary for this study.

In order to adequately answer the research question under lockdown restrictions, a brief app walkthrough was also undertaken to outline the various ways in which English learning can be achieved through WeChat. Furthermore, because individuals outside of China may not be familiar with WeChat or its respective functions, the walkthrough method provides an in-depth analysis of the WeChat application, thus making this research accessible to readers outside of China. The app walkthrough implemented in this study followed the template of Light, Burgess, and Duguay (2018) and complemented the data gathered from the ethnographic subjects in this study, the Chinese SA students. From a symbolic interactionist perspective, the app walkthrough ultimately details how some of the objective core features of WeChat can adopt symbolic meanings as English learning functions.

### Sampling and interview process

The data for this research were gathered in early 2021, and therefore, in order to protect the safety of the researcher and interviewees, the interviews were conducted online via Microsoft Teams and WeChat. The author’s university accepts a small group of international students from China every semester, and these SA students form the majority of the interviewees. The remaining interviewees were Chinese native students who attended university full-time and have experience learning English as a second language in Ireland. After contact was initiated with the participants, a consent form was provided to each participant, which outlined how the data they provided during the interview would be used. Semi-structured interviews were chosen for this study as they allowed for a free-flowing interview in which the interviewees were able to provide in-depth answers and express themselves freely (Flick et al. 2004).

While there are potential pitfalls and issues with qualitative data, Weiss (2004: 46) notes the importance of cooperation in receiving reliable information and ensuring a smooth interview, “while full cooperation cannot always be achieved, cooperation is likely to be maximized by an interviewer who is respectful and friendly, yet task-focused.”

### Data analysis

Most of the interviews were recorded using Microsoft Teams, and the interviews that took place on WeChat were recorded using a voice recording app. The data recorded were stored safely and password protected in order to maintain confidentiality.

Once the interviews were concluded, the dialog from the interviews was transcribed. Flick et al. (2004: 249) state, “transcripts are needed to make fleeting conversational behavior permanently available on paper for scientific analysis.” The audio from recorded meetings on Microsoft Teams is automatically transcribed, thus making the transcription process easier.

Once the transcriptions were completed, the coding process began using highlighting tools and in vivo coding. First-stage coding involved sorting topics by main headings, while second-stage coding involved sorting by subheadings (Dawson 2019). The utilization of coding made the data analysis section more straightforward to compose and allowed for accurate and relevant data as well as reoccurring themes to be highlighted within the raw data.

## Findings

### Demographics

This study aims to understand how Chinese SA students use WeChat for the symbolic purpose of EFL learning while exploring which particular features of WeChat are beneficial to one’s English learning. Furthermore, the study also aims to investigate the relationship between English proficiency and digital social capital on WeChat.

**Fig. 2:**
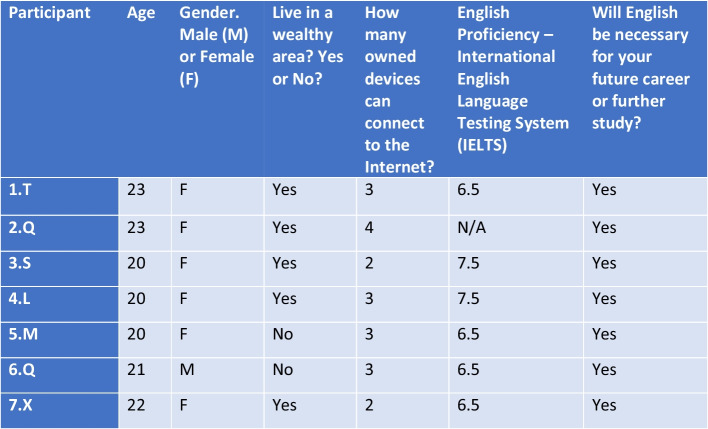
Table of demographics

Findings from the interviews were subsequently compared and contrasted with previous studies discussed in the literature review. The lack of gender diversity within the sample could be attributed to the fact that most Chinese SA students within this particular Irish university over the past 3 years have studied majors relating to education, and this is a field predominantly dominated by females (Fig. 2). Case in point, Zhang (2018) notes that in 2016, the class of graduates at East China’s Normal University (one of the biggest teacher training colleges in China) had a male-to-female ratio of 1–2.06.

Five of the seven interviewees noted that they lived in wealthy areas; however, three of these interviewees still described the English proficiency in their areas as “not good” or “bad.” However, this sentiment was mostly in reference to the older generations, as they are generally not able to speak English at all due to the fact that English education was outlawed within China in the past, particularly during the Cultural Revolution of 1966–1976 (Silver et al. 2002).

Most of the interviewees in the sample studied majors at an either undergraduate or postgraduate level related to English teaching or English translation. The exception in the sample was a Chinese student (2.Q) who had been a resident of Ireland for the past 5 years. Furthermore, all of the students had spent time studying abroad in an English-speaking country to some extent (a minimum of one semester). Moreover, the interviewees, due to their SA experience and background, represented an outlier group with higher proficiency in English than what would be considered average in China, as the lowest IELTS score among the interviewees was IELTS 6.5 and the highest was IELTS 7.5, respectively. To put this into perspective, Liao (2019) states that 5.72 was the average IELTS score among Chinese students within China in 2018.

Therefore, the sample does not reflect the realities of average university students in China as the sample consists of SA students who have specialized in English and inherently have access to more resources (Xu 2022). Furthermore, all seven interviewees desired to pursue further study or work in English-speaking countries. Hence, this should be taken into account while interpreting the findings.

### WeChat usage by Chinese study abroad students

The primary purpose of this subsection is to outline how often the participants use WeChat, and particularly how often they use WeChat for the symbolic purpose of EFL learning.

Based on previous research, a large-scale survey in China found that 64% of people use WeChat every day, and 39% of the students in the survey used WeChat to exchange information about studying (Cheng and Dong 2017). According to Shi et al. (2017: 19), in total, “approximately 50% of users (in China) use WeChat for at least 90 min a day.” Furthermore, despite the gender disparity in this study presenting potential drawbacks, Hou et al. (2020: 1822) found that “there is no difference between male and female students for WeChat learning engagement.”

**Fig. 3:**
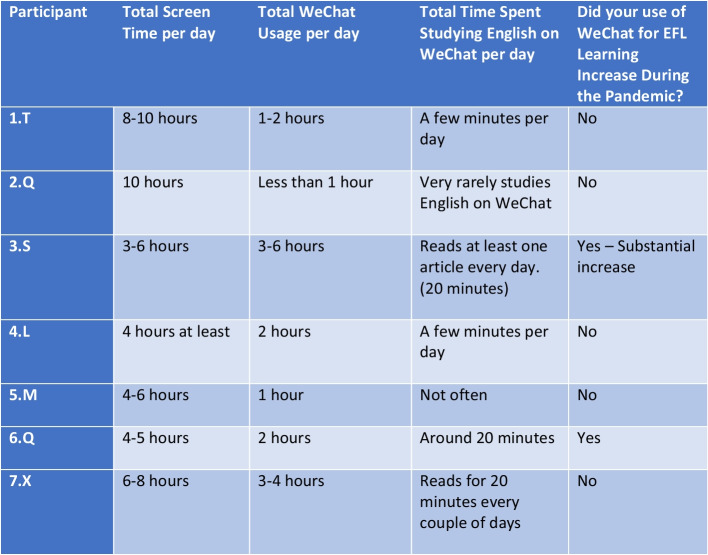
WeChat usage

All seven interviewees in this study stated that they use WeChat daily. Moreover, among the two participants with the lowest usage times (one hour or less), WeChat remained an integral part of their everyday lives due to the instant messaging aspect and keeping in touch with friends and family (Fig. 3). On this point, participant 6.Q stated, “my grandparents are on WeChat, so if I want to talk to them, I would just video-call them on WeChat.” Furthermore, despite participant 5.M stating that they use WeChat for only one hour per day, their usage was still characterized by frequently checking the app multiple times daily; “I don’t actually spend a long time on WeChat, but I do open the app for a few seconds quite regularly just to check my messages and moments. Actually, I do this a lot.”

As for using WeChat for the symbolic purpose of EFL learning, the interviewees’ opinions were split in this regard. While four out of seven interviewees engage with English content daily on WeChat, the remaining three use WeChat more sporadically for EFL learning purposes. However, this sporadic usage was characterized by the three interviewees indicating their general disinterest in English studying during their free time unless it was in “preparation for an exam.”

Interviewees were also split regarding whether or not their use of WeChat for EFL purposes increased during the pandemic, with only two interviewees indicating that the pandemic afforded them an opportunity to use WeChat for EFL learning further. One interviewee in particular stated,

*“Yes, actually. Uhm - pandemic time is my, you know, I would say peak time for me to learn English on WeChat because that's the time when I started to realize how helpful WeChat was. Like there are really a lot of resources which are free and which are quite beneficial for you to learn English.” (Interviewee 3.S)*

Meanwhile, another interviewee indicated why their usage did not increase,

*“No, I did not learn English on WeChat more often during this pandemic time. Actually, I used other learning apps, but I don’t know, I don’t really think of WeChat when I want to study.” (Interviewee 7.X)*

The account from participant 7.X above demonstrates that even though all interviewees indicated that they had both used WeChat to learn English and perceived WeChat as a useful English learning resource, not all participants fully engaged with the symbolic meaning of WeChat as an English learning tool. In a further example, participant 2.Q stated,

*“For Chinese people, we mainly use WeChat, you know to contact, to talk with people. And you have all your friends on WeChat and this is not easy to use one app both for studying and communicating.” (Interviewee 2.Q)*

2.Q’s sentiment that WeChat should be perceived more rigidly as purely a social media app was repeated by three other participants (1.T, 4.L, and 7.X), and when asked what features could be implemented within WeChat to assist English learning, 1.T replied, “it does not really need any changes. WeChat is a social media app, not a learning app.”

This complex perception of WeChat is echoed in Cheng and Dong’s (2017) study, who found that while students in both China and Sweden held positive attitudes toward the use of apps and mobile learning regarding learning languages, students did not hold any beliefs that there was an obvious relationship between WeChat and one’s learning interests. These findings demonstrate that while WeChat is regarded as beneficial to learning in previous research (Cheng and Dong 2017; Shi et al. 2017) and by all seven participants in this study, the influence of WeChat’s objective meaning as a social media app could represent a pertinent factor in whether or not it is used for EFL learning to a greater extent in the future. To elaborate on this point, the wider adoption of WeChat for learning English would require “routine cooperation, collective action, and shared experiences” (Segre 2019: 383). The subsequent sections explore why participants are hesitant to engage with WeChat’s symbolic purpose as an English learning tool.

### The walkthrough method

**Fig. 4:**
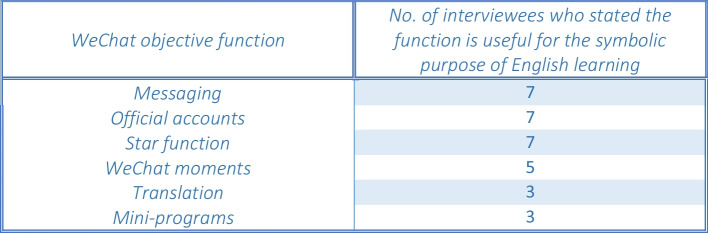
Objective functions of WeChat that participants use for EFL learning

This first section of the app walkthrough chapter will briefly discuss the objective features and mechanisms of WeChat that may be used for the symbolic purpose of English learning. In order to achieve this aim, the app walkthrough method by Light, Burgess, and Duguay (2018) was undertaken. Firstly, as an introduction, Fig. 4 details the WeChat features that the interviewees deemed useful for EFL learning purposes; however, it was only possible to discuss the top four most used functions in-depth within this study.

Regarding the validity of the walkthrough method, it should be noted that the walkthrough that the author conducted was underpinned by interviewee data, as during the interview process, interviewees were asked to share their screen with the author and detail how they access the features on WeChat which assist them with EFL learning. Furthermore, while the functionality of WeChat is comparatively much more limited outside of China, all seven interviewees stated that the features of WeChat that assisted them with EFL learning were unaffected while in Ireland. Furthermore, the author had access to all of the WeChat features the interviewees identified and was, therefore, able to conduct the app walkthrough accurately.

When discussing the “vision” or “objective” meaning of an app, it involves the “purpose, target user base, and scenarios of use” (Light et al. 2018: 889; Chen et al. 2020: 3). As clearly referenced in WeChat’s name and its emphasis on chatting, WeChat represents a social media app that’s objective vision is intended to connect friends and family members. Since its inception, WeChat has evolved its image into an “all-purpose app” that is not “just” social media (Montag et al. 2018: 2); however, the features that have contributed to WeChat’s image change are those provided by third parties such as “official accounts” or “mini-programs” and must be consciously searched for/downloaded by users. This demonstrates that users have considerable agency in regard to diverting from WeChat’s objective meaning as a social media app and consequently shaping their desired subjective meaning of WeChat or its respective functions.

**Fig. 5:**
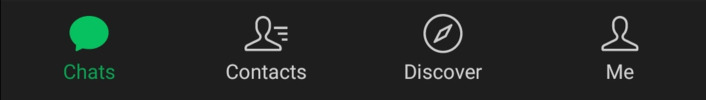
Four primary pages of WeChat

There are four main pages on WeChat, which can be seen in Fig. 5, including Chats, Contacts, Discover, and Me. Two of the four pages (Chats and Contacts) are concerned with messaging and connecting with friends, further illustrating that the main premise and objective vision of WeChat is chatting. Therefore, within the scope of symbolic interactionism, using WeChat for English learning purposes is a symbolic meaning given to the app and not part of its central vision or intended meaning (Segre 2019). An in-depth view of WeChat’s beneficial EFL learning features that the Chinese SA students highlighted will be discussed in the proceeding sections.

#### Official accounts

Within the findings, one feature that the interviewees unanimously regarded as beneficial to English learning was “official accounts.” Official accounts on WeChat upload articles and are described as “a China-based marketing platform that acts as a complete brand hub to gather followers, send them targeted content, and push them marketing and service notifications” (Wechatwiki 2019).

Official accounts allow users to construct a personal version of the WeChat app and engage with content that users must consciously search for depending on their preferences. On this point, official accounts are dedicated to a wide variety of interests, such as news, cooking, celebrities, and education.

When asked about the effectiveness of official accounts as an EFL learning feature, one of the interviewees stated,

*“Yeah, so I subscribed to a lot of official accounts for learning English, like there are many, many official accounts which are, you know, professional in teaching English. Like there are people who teach you how to read The Economist in a more efficient way, and that’s for free. So yeah, I often read some official accounts’ articles to learn English.”* (Interviewee 3.S)

**Fig. 6:**
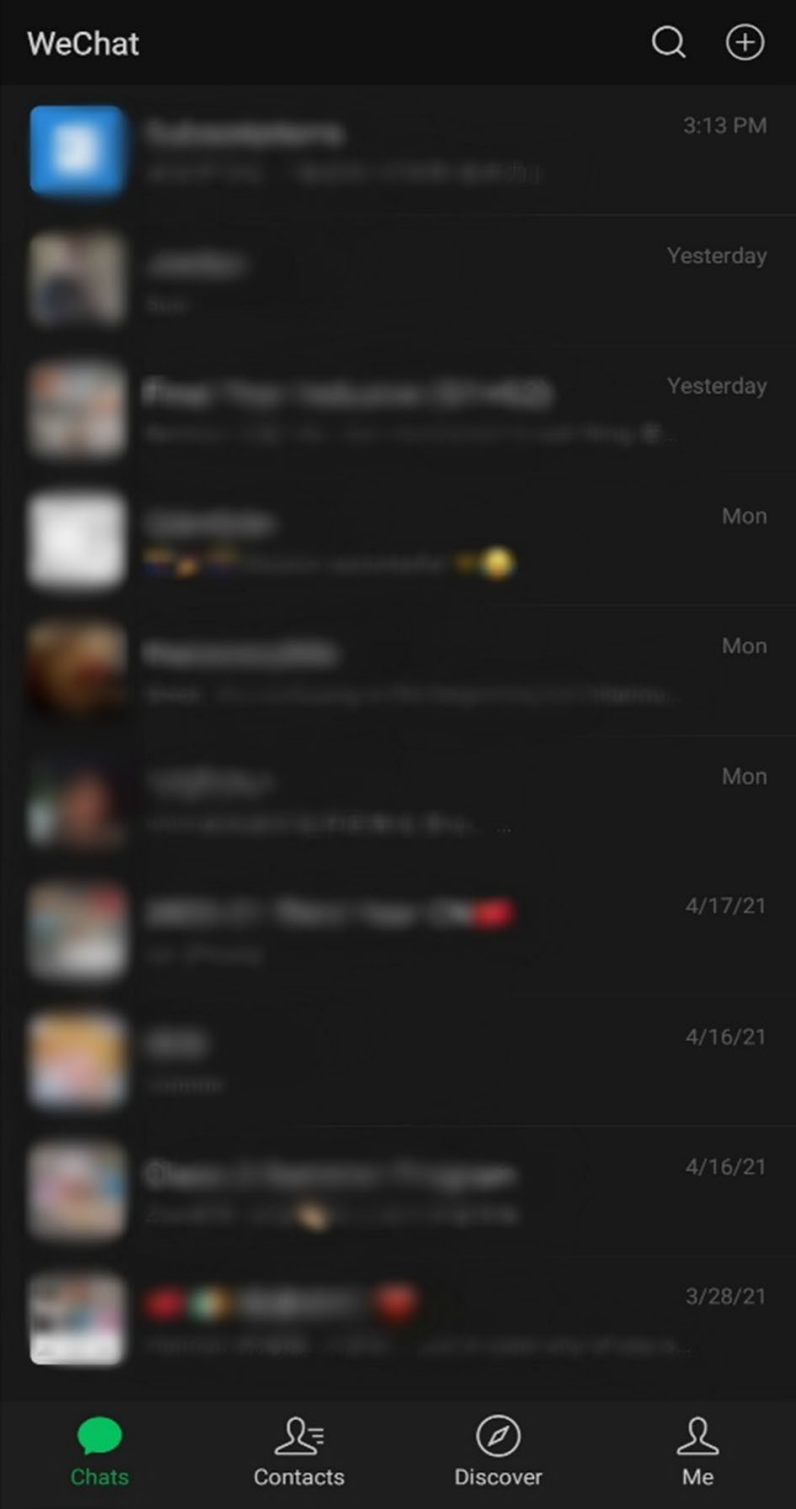
Home screen of WeChat. Note: The search symbol on the top right-hand side (first on the left) can be used to search for features throughout the app

**Fig. 7:**
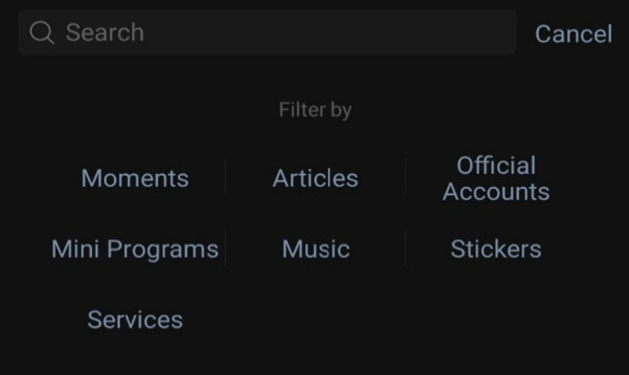
Upon selecting the search function, users can filter their searches

**Fig. 8:**
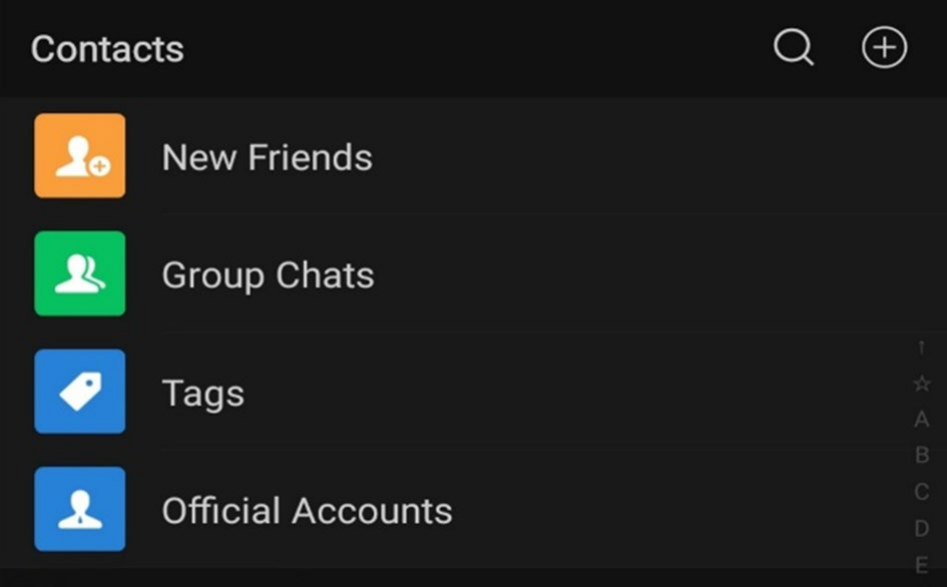
Within the contacts page, users can find official accounts

**Fig. 9:**
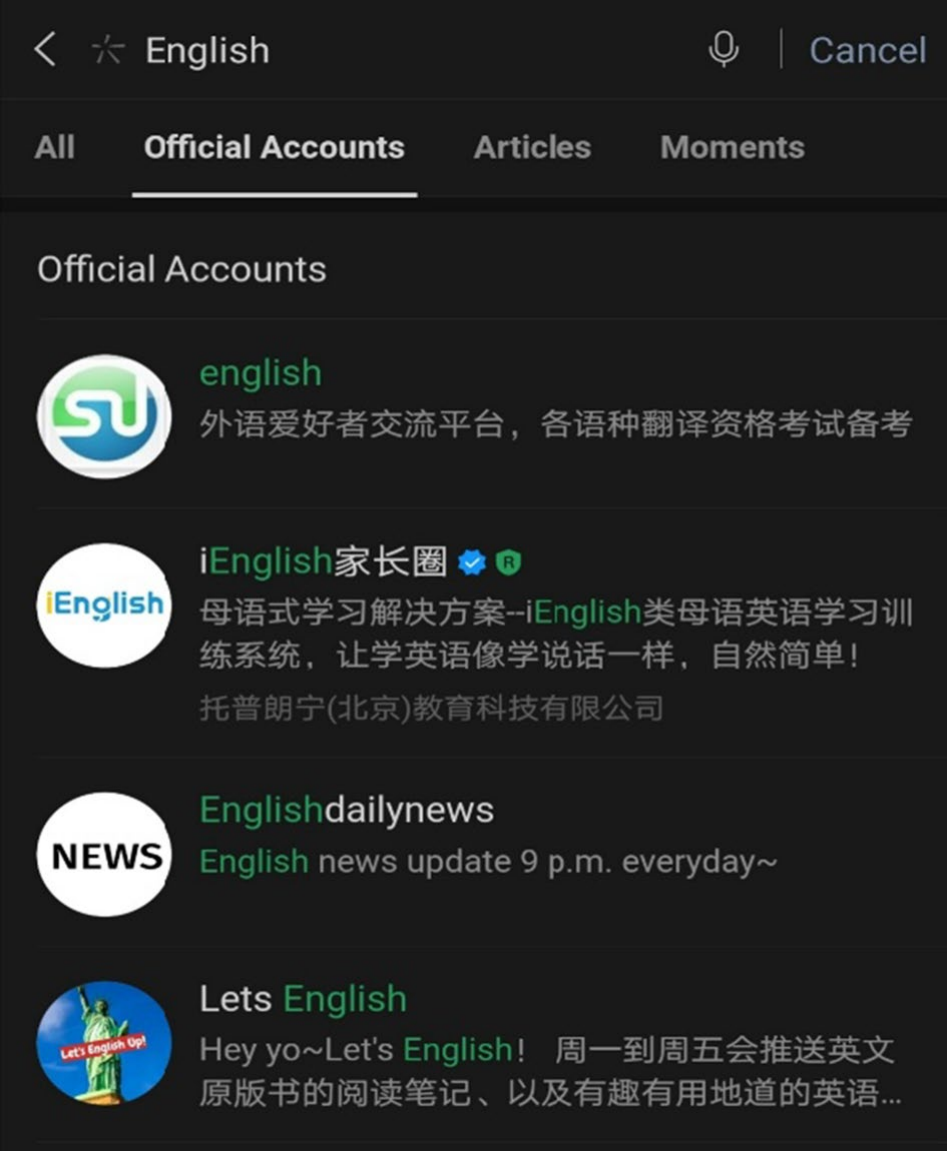
Results from searching for “English”

In order to find a particular official account, there are several methods, for example; users must already know the name of the account, type in a keyword(s) that relates to what they are looking for in the search, a friend shares the account with them, or users scan a QR code which leads to the account’s page (Figs. 6, 7, 8, and 9).

One interviewee indicated the method she uses to find EFL official accounts,

*“You can probably (search) for ‘English learning accounts’ and ‘English blogger,’ and you (can) probably see the recommendations and see which official account they recommend and just follow them. I think that’s the best way.” (Interviewee 2.Q)*

**Fig. 10:**
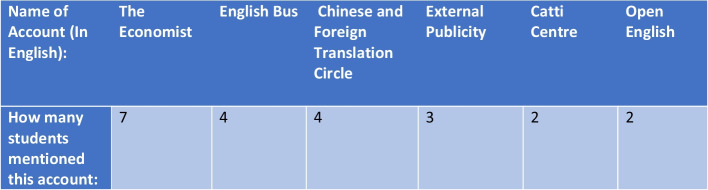
A table of EFL official accounts mentioned by interviewees (a minimum of 2 times)

Due to the presence of official accounts, WeChat offers users a free and simplistic way to engage with English content on a daily basis. For example, the official account “The Economist” translates news stories from Chinese into English and helps English learners develop a wider vocabulary. All interviewees cited this particular account as a useful resource for learning English as seen in Fig. 10.

The findings from this section highlight that official accounts are regarded as highly beneficial to EFL learning among all participants. As previous research suggested, the effectiveness of WeChat official accounts for English learning has only been discussed narrowly in regard to tweet-based writing (Zhang et al. 2021). Moreover, due to the fact that the interviewees engage with official accounts privately with no need to perform in the view of “observers” (Goffman 1959: 22), no drawbacks or negative social connotations in regard to engaging with official accounts were alluded to by any participants.

#### The star function

The participants also noted the importance of using the star function in relation to official accounts. The star function allows for official accounts that users are particularly interested in to appear higher up on their timelines. As mentioned in Chen and Wang’s (2022) respective walkthrough of official accounts related to COVID-19 information, WeChat uses an algorithm that favors state accounts and verified accounts that mainly consist of celebrities and large brands; however, despite the WeChat algorithm favoring certain accounts and attempting to shape users’ subjective meaning of WeChat to a certain extent, users still retain agency as they have the final decision in regard to following a particular account. However, even if a user follows an official account of their own choosing, there is no guarantee that any of the content from that account will be seen by the user due to WeChat’s algorithm.

On this point, all seven interviewees noted that if they did not “star” EFL learning official accounts on WeChat, they would subsequently be lost in the shuffle among the other accounts they follow. Therefore, the star function highlights a feature that all participants use to retain user agency in shaping their own subjective meaning of WeChat as an English learning tool. One interviewee in particular stated,

*“Yeah, I use the ‘star’ function to highlight those useful official accounts which can access learning English, because if you don't ‘star’ it, it will be overwhelmed - you know it will be overwhelmed by other official accounts. So, if you ‘star’ it, it just shows on the top, and you never miss the articles it sends you.” (Interviewee 3.S)*

**Fig. 11:**
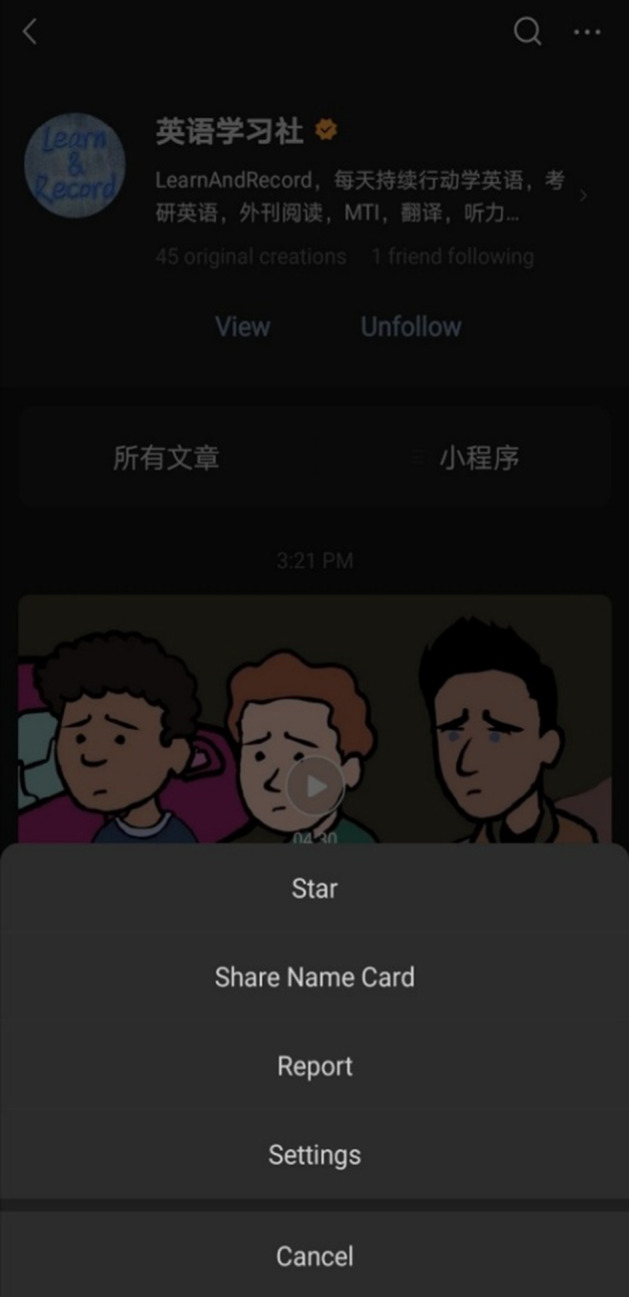
When users click on an official account, they are afforded the option to “star” it

While not reported in previous research, the star function is, therefore, necessary for WeChat users who wish to use WeChat to learn English, as all interviewees indicated that they had used the star function to prioritize EFL content (Fig. 11). Moreover, much like official accounts, the interviewees did not report any drawbacks or negative social connotations when using the star function.

#### Chatting for English learning

As mentioned in the literature review, Cheng and Dong (2017), Shi et al. (2017), and Wang and Crosthwaite (2021) explored the effectiveness of WeChat’s instant messaging features in relation to English learning. However, this section aims to provide greater detail in regard to the multitude of ways in which users can use WeChat’s objective chatting function for the symbolic purpose of EFL learning.

Within WeChat, on the screen labeled “chats,” users can view all of the conversations that they previously had with their friends. “Chats,” therefore, represents the main page of WeChat as it is the first screen that users are greeted with when they open the application. While chatting is WeChat’s objective meaning, all seven participants unanimously stated that they had used the chatting function of WeChat for the symbolic purpose of English learning at some point and perceived it as a useful EFL learning function.

**Fig. 12:**
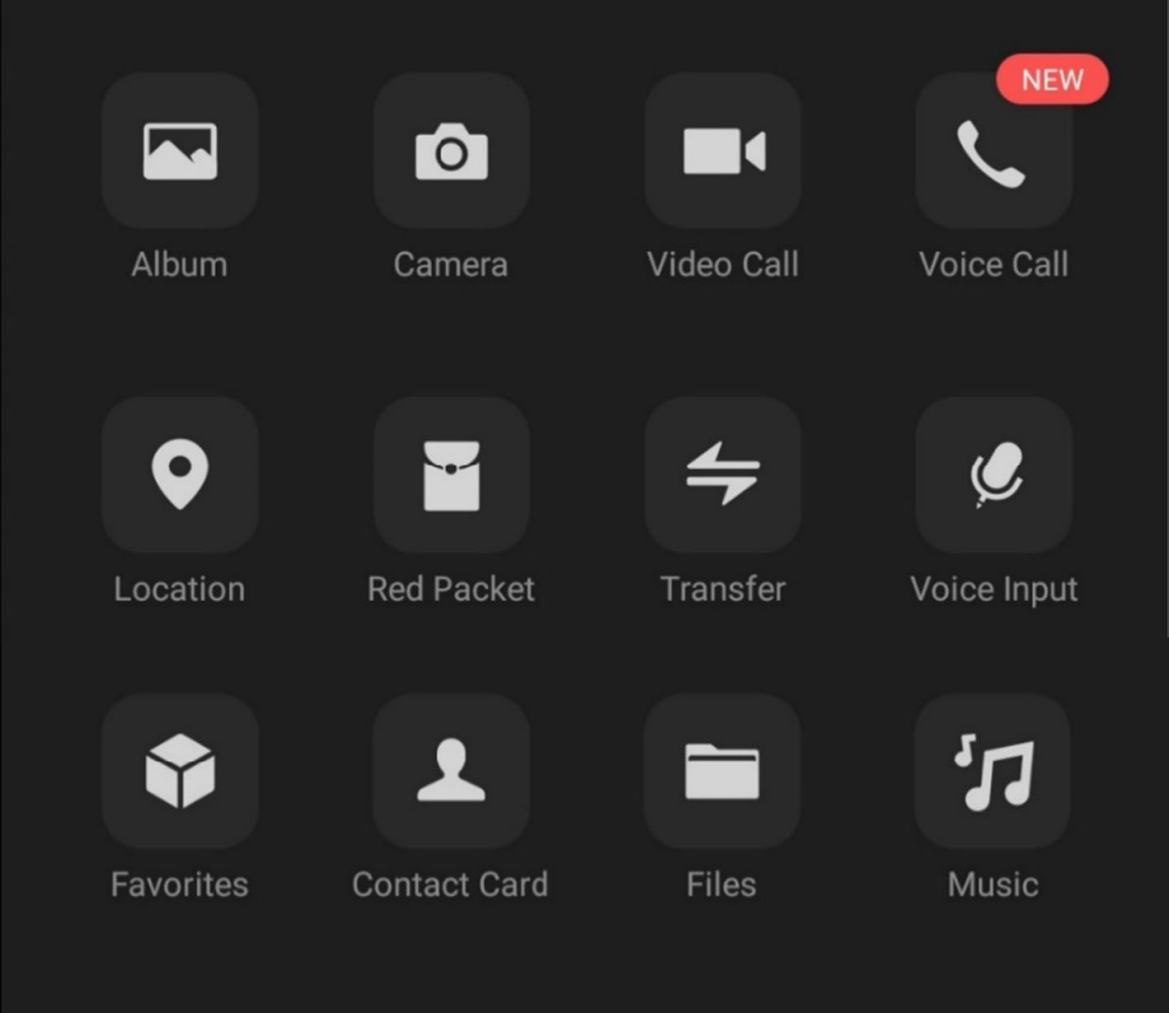
Features that WeChat users may use when chatting with others

**Fig. 13:**
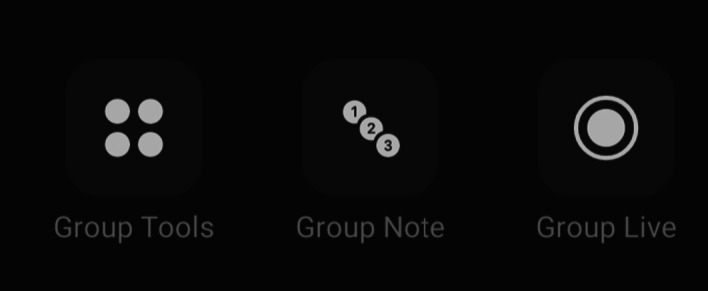
Extra features available while in group chats

As shown in Figs. 12 and 13, when users open a new chat on a freshly downloaded version of WeChat, they have a variety of objective functions to choose. One can send pictures, files, and voice messages to other users. Furthermore, users may conduct voice and video calls.

When describing the benefits of the instant messaging function for English language learning, one interviewee indicated,

*“Oh, there are lots of ways we can help each other on WeChat. We can message each other with questions, send resources, and if we have a foreigner friend - we can call them to practice English.” (Interviewee 1.T).*

Furthermore, three out of seven interviewees indicated that they are friends with their English teachers on WeChat and that their teachers use this platform to answer any questions the students pose and provide learning materials.

In the study conducted by Cheng and Dong (2017), 95% of students found that having a class group chat with a teacher present was beneficial for English learning, and within this study, when describing how her teacher used WeChat to contact her, one interviewee stated,

*“Due to Covid, the teacher could call me, carry out some activities and pass some information through the chat, and he would contact me (privately) a lot, but - he would mainly use our group to survey some information because it's more efficient.” (Interviewee 4.L)*

When asked if she communicates in the class group chat often, she replied,

“Not really. We are all very quiet in the group apart from the teacher. He is the one who speaks. Even if I had a question, I would not ask in the group, I may just message my classmate (privately).” *(Interviewee 4.L)*

Another interviewee who is a member of an English class group chat had a similar response when asked if they engage with the group chat,

“No, we tend to be shy. There are a couple of students who always speak, but it is a bit strange. I don’t know. The Chinese way is to be quiet. The students who speak must want us to think they are smart, but I don’t think so (laughs) - I just read the information the teacher sends.” (Interviewee 1.T)

Moreover, a further interviewee stated that the objective meaning of instant messaging for communicative purposes could distract the user from the symbolic purpose of learning English,

“And since many Chinese people use WeChat to communicate, sometimes you are easily bothered by others sending messages to you while you're learning English.” (Interviewee 3.S)

**Fig. 14:**
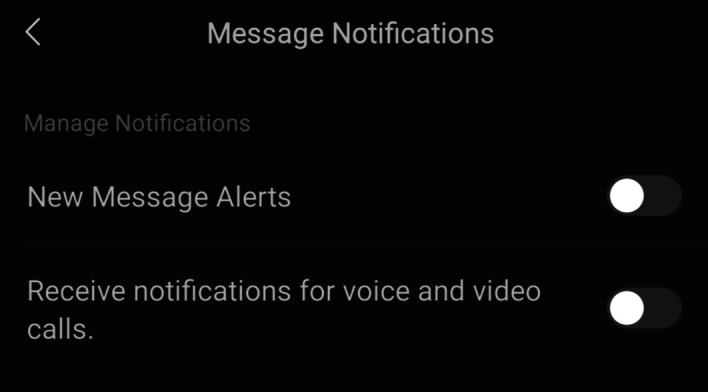
WeChat allows users to turn off their message notifications to prevent distraction

However, in order to address the issue raised by interviewee 3.S, users could turn off their message alerts during the time they allocate to English learning on WeChat and prevent the objective meaning of WeChat as a social media application from causing a distraction (shown in Fig. 14). Furthermore, the drawback indicated by 3.S could apply to all English learning applications (not just WeChat) as long as message notifications were enabled on their device.

While both 1.T and 4.L indicated that group chats with their teachers present were useful, both interviewees admitted to not fully engaging with the group chats for the symbolic purpose of EFL learning and using it to their full potential. Furthermore, both interviewees refer to the possible Confucian connotations and “Chinese way” of quietness (Xie 2010; Li and Wegerif 2014) and respect for their teacher (Littrell 2005; Zhu and O’Sullivan 2022) as causing this lack of engagement. Moreover, interviewee 1.T indicated that the students who spoke and engaged with the English language class group chat might be trying to perform themselves in the group in order to be perceived as smarter and more impressive.

From a symbolic interactionist perspective, despite regarding them as a useful EFL learning resource, the interviewees’ symbolic meanings and engagement with group chats were influenced by their perception of other users’ actions (Chen et al. 2020). Furthermore, 1.T indicated that her classmates’ performances of their identity in the group chat had a negative influence on her (the observer) which resulted in her referring to the performance as “strange.” These negative connotations of performing the self as an EFL learner resulted in some participants not fully engaging with WeChat for the symbolic purpose of EFL learning (McCay-Peet and Quan-Haase 2016).

However, within the context of privately messaging friends or classmates, as stated by interviewee 4.L, there were no such negative connotations, thus demonstrating the pertinence of context (private messaging or group chats), positive experience (distraction caused by others’ messages), and associating negative connotations with performing the self (showing off to appear smart) in regard to fully engaging with the instant messaging aspect of WeChat for English learning.

#### WeChat moments as an EFL learning tool

**Fig. 15:**
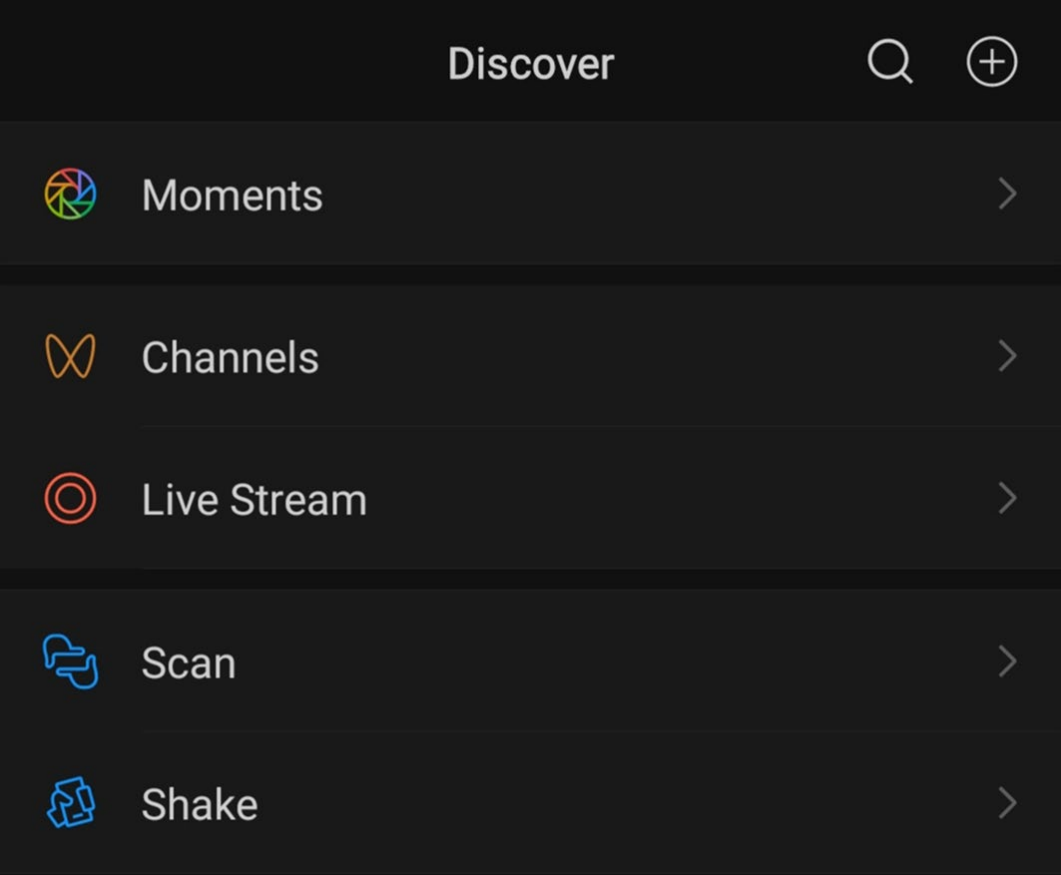
WeChat moments are found on the discover page

**Fig. 16:**
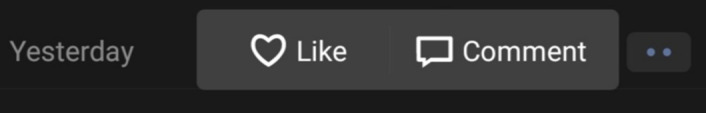
WeChat moments are viewable to all of a user’s friends on WeChat, and users can like or comment on a moment

**Fig. 17:**
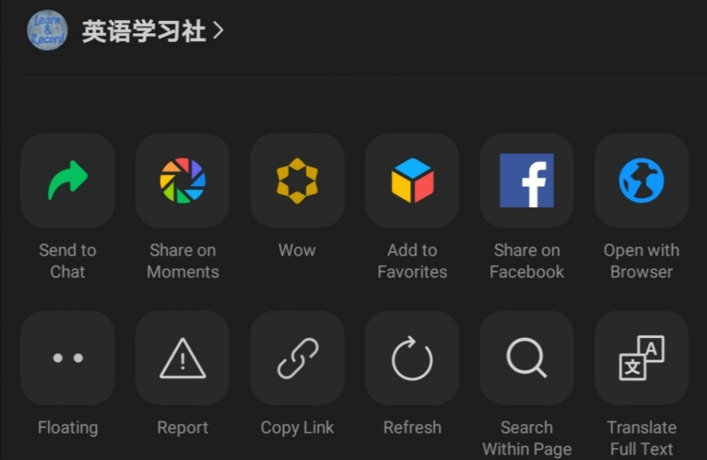
Users can share articles and learning resources on their moments

The final objective function of WeChat which has symbolic capabilities as an English learning feature is the ability to post “moments.” WeChat moments allow users to share text, pictures, media, and articles with their entire circle of friends, and users can interact with each other by either liking or writing a ﻿comment underneath a particular moment (Figs. 15, 16 and 17).

Five out of seven interviewees perceived WeChat moments as having promise as an English learning resource, and one interviewee stated,

*“Oh, I guess moments could be helpful too. I haven’t done this for English yet, but if I need help with an issue, I can post a moment asking my circle of friends for help - they can answer me under the moment.” (Interviewee 5.M)*

Another interview stated,

*“I also think moments can be used (for English learning). We can follow what our teacher posts, or we can share some information with our friends or ask questions.” (Interviewee 2.Q)*

Interviewee 1.T mentioned that certain English learning apps automatically post moments on users’ WeChat timelines that showcase their learning progress and state how many words they have learned on a particular day. While this practice allows English learning apps to advertise themselves on WeChat for free, one interviewee stated that the apps incentivize EFL learners to post moments on WeChat,

*“If you allow the app to post updates on your timeline, you earn coins which can be used in the app. If you collect enough coins, you can get the full app for free and unlock more functions.” (Interviewee 1.T)*

Therefore, publicly presenting oneself as an English learner on WeChat presents potential benefits across various EFL learning applications.

However, similar to the chatting function mentioned previously, posting WeChat moments has social connotations that involve users performing their English learning identity in view of observers. As Hou et al. (2020: 1823) state, “WeChat is a window for people to demonstrate themselves, so people would like to show their good sides to others.” Furthermore, according to McCay-Peet and Quan-Haase (2016: 200), the ability to openly present the self on social media directly translates to the engagement and “quality of user experience” that one has with a particular social media application. In terms of the interviewees’ attitudes toward posting English content via WeChat moments, their attitudes differed in this respect. When asked whether or not they would post content related to their experiences of learning English, one participant stated,

*“I don’t really like to share my learning, showing how many (English) words (I learned) today - And for me, I don’t, I don’t do that. I don’t know. It’s probably not too good ‘cause I don’t wanna show people, like OK, I’m doing this. I don’t know. Probably for some people, but not for everyone.” (Interviewee 2.Q)*

Another interviewee said similar,

*“I don’t post any English resources and ask questions on my moments. I know it’s not - it’s not very good (to not use this feature). I just read my friend’s and teacher’s moments instead when they speak about English stuff.” (Interviewee 5.M)*

However, one interviewee mentioned that others posting about their English learning experiences motivated her to improve,

*“I never think it is bad because even - even if the people are just bragging about the certificates, I just think, well, they are really somebody and – and I think I like to see people share their experiences because I could see more about what my peers are doing, so I won’t be left behind, yeah.” (Interviewee 4.L)*

In terms of uploading moments on WeChat that are merely written in the English language and which do not mention their learning experiences, four out of seven interviewees were hesitant to perform this action due to their friends and family in China. In order to explain, one notable aspect of Confucian culture is that parents are shown a greater amount of respect, and there are greater expectations for children to live close to their parents and take care of them in their old age (Fan 2006). This tradition in China is called filial piety (xiào孝), and a common Chinese phrase, when translated to English, reads as, “raising a child offers (the parent) insurance in old age” (yǎng ér fáng lǎo养儿防老). The main aspects of filial piety consist of respect for parents, obedience, loyalty, material provision, and caring for parents when they are older, and this can also extend to older family members (Zhan and Montgomery 2003; Yeh et al. 2013). Furthermore, despite the interviewees’ status as SA students and being regarded as neoliberal subjects in the literature (Xu 2022), a number of interviewees still exhibited Confucian tendencies in regard to posting on WeChat.

On this point, participant 7.X noted that while she does sometimes post moments in English due to the fact it is “more simple to express certain words” in the English language rather than Chinese, she also noted,

*“If I write in English, my mom or my cousin will ask me what the sentence means, and they will be like, ‘why are you writing in English? You are Chinese’. So, I don’t write in English really because of that.” (Interviewee 7.X)*

Furthermore, when interviewee 7.X was asked whether or not her parents or relatives could just use the translation function of WeChat, she noted, “Yeah, that’s true. I don’t know. I just think it is more important that they understand, so I should just write in Chinese, yeah.”

Another interviewee also stated the importance of her family being able to understand her WeChat posts,

*“It’s just not good, ‘cause their English is not too good, and you always choose something they don’t understand. I have a cousin, and I think I posted just one sentence in English or something, and then he commented saying, ‘Why don't you speak Chinese? It's really annoying.’ So, after that, I just stopped there.” (Interviewee 2.Q)*

In regard to having an impact on their “observers” (Goffman 1959: 22), Chinese SA students draw criticism from family members when they perform their identity as English language learners. This has consequently caused some interviewees to stop posting in English on their WeChat moments despite identifying that WeChat moments are helpful for EFL learning. Therefore, interviewees were found to not fully engage with WeChat moment’s symbolic meaning due to not freely performing themselves in front of observers (McCay-Peet and Quan-Haase 2016). Instead, participants preferred to read their English teachers’ WeChat moments comprising articles or learning materials they publicly shared. This subsequently underpins symbolic interactionist theorizing by Jones and Volpe (2011: 426), who stated that one’s social networks “generate meanings” for users on a particular app.

**Fig. 18:**
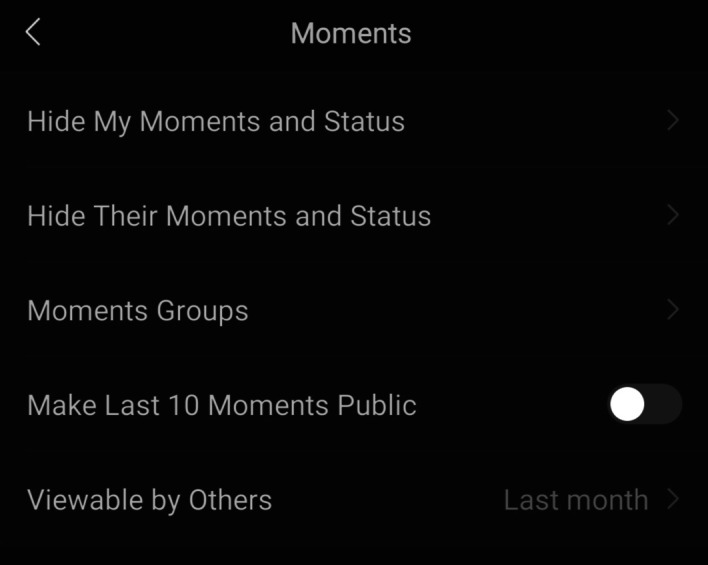
Option to only showcase moments to a selected group of people

However, while not mentioned by the participants in this study, as seen in Fig. 18, one has the option to hide WeChat moments from other users by preselecting which users can see their moments, and this particular feature was mentioned in the study by Huang et al. (2020).

As a practical suggestion for Chinese EFL learners, rather than users allowing their symbolic use and engagement of WeChat moments for the purpose of learning to be affected by notions of family influence, digital social capital (as will be mentioned in the next section), negative experience (family influence was subsequently regarded as negative in this study), and performance of the self (participants in this study were hesitant in posting moments in English due to the factors mentioned), users could instead choose which observers can view their WeChat moments.

Therefore, to conclude the walkthrough section and RQ.1, the participants identified instant messaging, official accounts, the star function, WeChat moments, mini-programs, and the translation function as objective WeChat features which could be used for the subjective purpose of learning English. To touch upon RQ.1a, despite a number of participants highlighting issues that apply to social media as a whole regarding EFL learning on WeChat, such as the potential for distractions, negative social connotations, and an algorithm controlling which accounts are recommended to users, these represent drawbacks with WeChat’s objective meaning. However, WeChat has a fluid role and allows the user to retain agency in controlling their perceived subjective meaning of WeChat as an English learning tool. On this point, users must make conscious decisions to deviate from WeChat’s objective vision and optimize it for the symbolic purpose of EFL learning. To elaborate, WeChat allows users to star their favorite official accounts to ensure they see the content they want, disable message notifications to prevent distractions, and limit which users can see their English-related WeChat moments. Therefore, this walkthrough of WeChat highlights features of WeChat that can be used for English learning that have not yet been explored in previous research. Moreover, this study also details how to avoid the various socio-material drawbacks present within a number of WeChat’s objective features when used for the symbolic purpose of EFL learning.

### English proficiency’s link to digital social capital

As already established in the literature review, “English is a type of cultural advantage enjoyed by students of privileged backgrounds in China” (Liu and Chiang 2019: 10). In regard to exploring how English proficiency is linked to higher-class and socioeconomic status, Bourdieu’s theory of social capital and Julien’s (2015) concept of digital social capital will be discussed. All seven interviewees drew links between English proficiency and socioeconomic status, with the underlying themes being that, in order to have high proficiency in English, an individual or their family must be wealthy, have spent time abroad in an English-speaking country, have paid for a private tutor/private classes, or have attended school in a Tier-1 city in China. On this point, when asked whether high proficiency in English is related to wealth in China, one interviewee responded by stating,

*“So yeah, higher proficiency does have something to do with, you know, wealth in China because the people - eh, I would say the developed areas have those wealthy people who can access a lot of resources to improve their English level, like there are many international schools in highly developed areas. Also, they can access many foreigners who can be their friends or their teachers like yeah. So - yeah, I think it's highly related.” (Interviewee 3.S)*

This phenomenon of wealth being advantageous for English proficiency and education in China can be explained by Bourdieu’s theory of social capital, as the education system assumes the “possession of social capital.” Therefore, students from lower classes or those who have less access to resources are ultimately at a disadvantage which in turn leads to it being “very difficult for (them) to succeed in the education system” (Sullivan 2002: 145).

Considering that all the interviewees agree that high proficiency in English is linked to wealth and social capital in China, it is now pertinent to determine whether performing one’s English proficiency on WeChat is regarded as a form of digital social capital. When discussing digital social capital, Julien (2015: 365) indicated that the exchange of digital social capital online via memes, posts, or pictures, “cement the things exchanged as permanent tokens of group membership, and the exchanges also reproduce the group.” WeChat comprises objective functions such as messaging and posting WeChat moments that involve engaging with others and performing the self. Moreover, they also have symbolic meanings as EFL learning features, according to the participants in Fig. 4.

In order to determine whether interviewees believed that English learners’ performances of their identity constituted forms of digital social capital on WeChat, interviewees were asked about their perception of publicly posting content related to learning English. One interviewee stated,

*“We don’t usually post our English learning stuff on WeChat moments, yeah. I think maybe it’s because a part of us feels shy to do so. Like people know, people know that you are learning English, and it feels like you are showing off sometimes, as people would sometimes think like that, so yeah.” (Interviewee 3.S)*

Here 3.S admits that publicly performing their English proficiency online or merely mentioning their English learning is something that they fear may be interpreted as “showing off.” This sentiment was repeated by another interviewee who stated,

*“Even though we all learn English - if I saw a Chinese person write in English on their WeChat moments, I would think it is a little strange. Like they are trying to impress someone. We generally don’t do that.” (Interviewee 6.Q)*

Considering that all the interviewees established earlier that English proficiency has strong links with wealth in China, excerpts from interviewees 3.S and 6.Q indicate that English proficiency is used as a means to show off, or to try and impress others, thus demonstrating that it can indeed be interpreted as a form of digital social capital on the WeChat app and thus addresses RQ.2 in the process. Moreover, interviewee 1.T indicated that she negatively perceived her classmates who performed their role as English learners in front of others in order to appear “smarter” and referred to them as “strange.” By applying Julien’s (2015: 367) concept of digital social capital, the “desire of individuals for recognition motivates their distinguishing actions as they exist in social space.” While all the interviewees possess membership as EFL learners, they were able to recognize when other users in the same social group performed distinguishing actions on WeChat to make their digital social capital more apparent and achieve greater recognition. Case in point, the specific actions consisted of publicly performing their English proficiency or identity as an English learner for others to see. Hence, in the views of four out of seven interviewees, the performance of English proficiency should be confined to the “backstage” where no observers can see (Goffman 1959: 69) and should be a “suppressed” action in the words of Goffman.

This underpins previous findings by Yang (2007: 12), who found that an online performance of English proficiency is linked with “showing off” in China due to the links between English education and socioeconomic status. Moreover, the findings of this study which explore the negative aspects of possessing digital social capital are pertinent, as Jafari and Moharrami (2019: 8) indicate that the majority of previous research on digital social capital merely focuses on the positive outcomes and “prosocial,” “beneficial functions for sociability.”

Therefore, while five participants broadly regarded WeChat moments as helpful for EFL learning, by using the theoretical framework (Fig. 1), the interviewees associated performing their English proficiency and identity as English learners in front of “observers” with negative connotations of “bragging” which resulted from associations with digital social capital (Goffman 1959: 22). This consequently resulted in four out of 7 participants stating that one should not publicly perform as an English learner on WeChat. From a symbolic interactionist perspective, both the “meaning” that the participants attached to WeChat moments and their attitudes toward performing their identities was directly influenced by their social networks (Jones and Volpe 2011: 426). Therefore, to firmly address RQ.1a, due to potential observers creating negative meanings for participants regarding publicly performing as an EFL learner on WeChat, the participants did not fully engage with the WeChat moments feature for the symbolic purpose of learning English, as the ability to freely perform oneself on an app is directly linked to whether one engages with an app for a particular purpose (McCay-Peet and Quan-Haase 2016).

The findings, therefore, highlight that EFL learning on WeChat takes place amid a complex socio-material backdrop in which the English language is perceived as a form of digital social capital and results in connotations of higher class and showing off. This study advances the understanding of why Chinese SA students may or may not choose to fully engage with a particular feature of WeChat for the symbolic purpose of EFL learning and highlights the challenges that language practitioners must overcome regarding using WeChat as an EFL learning tool. Furthermore, along with digital social capital, the theoretical framework implies that filial obligations to obey and respect parents and older family members also prevented some participants from publicly performing their roles as EFL learners or fully engaging with WeChat moments as an English learning tool. However, while perceptions of digital social capital and filial obligations present deep-rooted societal challenges in regard to EFL learning on social media and WeChat in particular, the walkthrough section highlighted a number of WeChat features that users can use to circumvent the perceived negative social connotations associated with WeChat’s objective meaning as a social media application. Therefore, this article demonstrates how users can engage with WeChat’s symbolic meaning as an EFL learning tool to a greater extent, and future research in this area is encouraged.

## Conclusion

The research questions of this study were based on understanding how Chinese SA students use WeChat for the symbolic purpose of EFL learning while exploring what features of WeChat contribute to one’s English learning in particular. This study also investigated how English proficiency acts as a form of digital social capital on WeChat.

In order to answer the research questions, the study used a theoretical framework that incorporated theories of symbolic interactionism, Julien’s (2015) digital social capital, and Goffman’s (1959) presentation of self. Furthermore, the app walkthrough method by Light, Burgess, and Duguay (2018) was used to highlight the objective functions of WeChat that could adopt symbolic meanings as English learning functions. The identified functions were messaging, official accounts, the star function, moments, mini-programs, and the translation function.

Regarding English proficiency representing a form of digital social capital on WeChat, due to the links between English proficiency and higher-class status in China, participants identified that instant messaging and WeChat moments represented platforms from which users could perform in front of “observers” (Goffman 1959: 22). Therefore, the majority of the participants associated those Chinese EFL learners, who publicly performed “distinguishing actions” on WeChat in the hopes of receiving “recognition,” with negative connotations such as “bragging” or “showing off” their higher status as English learners (Julien 2015).

Hence, by using the theoretical framework, despite the participants having positive perceptions of WeChat’s symbolic effectiveness for English learning, due to negative experiences of digital social capital, family influence (as a result of Confucian influence), and context, the participants consequently possessed negative attitudes in regard to performing their role as EFL learners on the instant messaging and moments features of WeChat. Furthermore, as McCay-Peet and Quan-Haase (2016) indicated, the ability to freely perform oneself on an app was necessary to engage with the app for a particular purpose. Therefore, participants in this study did not fully engage with the instant messaging and moments features for the symbolic purpose of learning English.

By comparison, due to fact that the other objective features of WeChat, such as official accounts, mini-programs, and both the star and translation functions, did not require participants to perform their identity as an English language learner in front of observers on WeChat, these particular features did not result in any negative social connotations.

This study, therefore, demonstrates that while participants all perceived WeChat as beneficial to EFL learning, WeChat’s objective meaning as a social media app presents challenges for Chinese EFL learners regarding fully engaging with all of WeChat’s objective functions for the symbolic purpose of learning English. However, as the walkthrough section highlighted, WeChat allows users to circumvent a number of the potential socio-material drawbacks that the participants outlined, thus indicating WeChat’s potential wider adoption as a language learning tool. Therefore, the findings of this study are of particular pertinence to language practitioners and Chinese SA students who incorporate WeChat into their EFL teaching/learning practices, and the findings should act as a starting point for future research to explore the effectiveness of WeChat’s functions for EFL learning to a greater extent.

### Limitations

Due to the small sample size, this study can in no way offer a generalization of Chinese SA students who use WeChat. The sample also did not include any truly disadvantaged Chinese SA students as this would be difficult to contact; therefore, the findings may be considered one-sided.

### Future research

Since this study focused solely on Chinese SA students within the university, future research could focus on other levels of education, such as secondary schools or language learning among adults within China. While highlighted in the walkthrough section, future studies could also explore the effectiveness of using the “hide moments” function on WeChat and determine whether this feature prevents the negative social connotations cited within this article in regard to WeChat moments.

## Data Availability

All data gathered from the participants’ recorded interviews are safely stored on the author’s hard drive. Data supporting the findings will not be shared for ethical reasons in order to protect the participants’ privacy.

## Abbreviations

CE: College English
EFL: English as a Foreign Language
F: Female
IELTS: International English Language Testing System
M: Male
N/A: Not available
No.: Number
SA: Study abroad
